# Sulconazole Induces PANoptosis by Triggering Oxidative Stress and Inhibiting Glycolysis to Increase Radiosensitivity in Esophageal Cancer

**DOI:** 10.1016/j.mcpro.2023.100551

**Published:** 2023-04-17

**Authors:** Lu-Xin Liu, Jing-Hua Heng, Dan-Xia Deng, Hui Zhao, Zhen-Yuan Zheng, Lian-Di Liao, Wan Lin, Xiu-E. Xu, En-Min Li, Li-Yan Xu

**Affiliations:** 1 Guangdong Provincial Key Laboratory of Infectious Diseases and Molecular Immunopathology, Institute of Oncologic Pathology, Shantou University Medical College, Shantou, Guangdong, China; 2 The Key Laboratory of Molecular Biology for High Cancer Incidence Coastal Chaoshan Area, Department of Biochemistry and Molecular Biology, Shantou University Medical College, Shantou, Guangdong, China; 3 Guangdong Esophageal Cancer Research Institute, Shantou Sub-center, Cancer Research Center, Shantou University Medical College, Shantou, Guangdong, China

**Keywords:** sulconazole, PANoptosis, oxidative stress, glycolysis, radiosensitivity, esophageal cancer

## Abstract

Esophageal cancer is the seventh most common cancer in the world. Although traditional treatment methods such as radiotherapy and chemotherapy have good effects, their side effects and drug resistance remain problematic. The repositioning of drug function provides new ideas for the research and development of anticancer drugs. We previously showed that the Food and Drug Administration–approved drug sulconazole can effectively inhibit the growth of esophageal cancer cells, but its molecular mechanism is not clear. Here, our study demonstrated that sulconazole had a broad spectrum of anticancer effects. It can not only inhibit the proliferation but also inhibit the migration of esophageal cancer cells. Both transcriptomic sequencing and proteomic sequencing showed that sulconazole could promote various types of programmed cell death and inhibit glycolysis and its related pathways. Experimentally, we found that sulconazole induced apoptosis, pyroptosis, necroptosis, and ferroptosis. Mechanistically, sulconazole triggered mitochondrial oxidative stress and inhibited glycolysis. Finally, we showed that low-dose sulconazole can increase radiosensitivity of esophageal cancer cells. Taken together, these new findings provide strong laboratory evidence for the clinical application of sulconazole in esophageal cancer.

## Highlights

• Sulconazole has a broad spectrum of anticancer effects.
• Sulconazole induces PANoptosis of esophageal cancer cells.
• Sulconazole triggers mitochondrial oxidative stress and inhibits glycolysis.
• Sulconazole increase the radiosensitivity of esophageal cancer cells.

## Graphical Abstract

**Figure:**
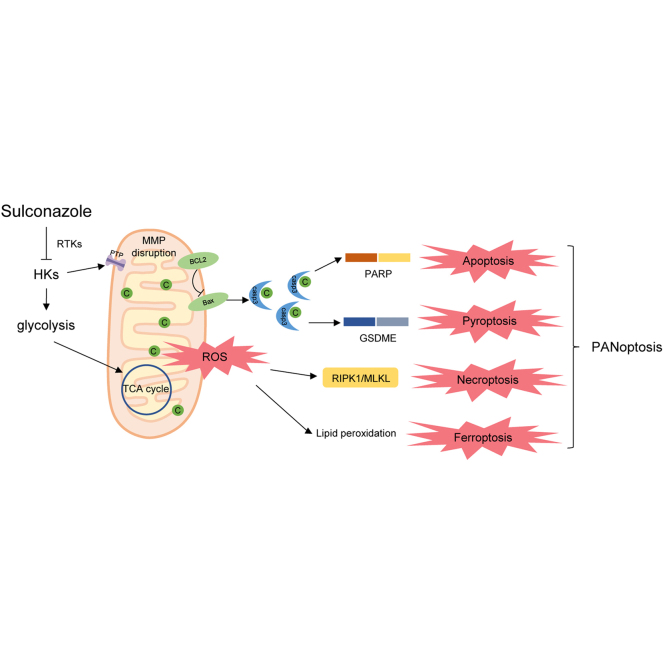

## In Brief

This article reports that sulconazole induces PANoptosis, which is a combination of cell apoptosis, pyroptosis, necroptosis, and ferroptosis, in esophageal cancer cells. Mechanistically, sulconazole triggers oxidative stress and inhibits glycolysis *via* downregulating HKs and inhibiting the PI3K/AKT, MEK/ERK, STAT3 pathways. Finally, sulconazole increases radiosensitivity of esophageal cancer cells. This study provides experimental evidence for the clinical application of sulconazole.

Esophageal cancer ranks seventh in incidence and sixth in mortality worldwide and is mainly comprised of esophageal squamous cell carcinoma and esophageal adenocarcinoma (1, 2). There are multiple choices for esophageal cancer treatment, including endoscopic management, surgery, chemotherapy, radiotherapy, and immunotherapy, depending on the tumor node metastasis stage combined with patient characteristics (3). Early-stage esophageal cancers are adaptable to endoscopic resection, whereas locally advanced and nonmetastatic tumors are suitable for surgical management. Neoadjuvant, perioperative chemotherapy, radiotherapy, or chemoradiotherapy may be more suitable for patients with ≥ T2 tumors; immunotherapy trials are still on going. However, the 5-year survival rate of esophageal cancer patients is less than 20% (4). The low survival rate can be attributed to the complex biological behavior of tumor cells, which show great resistance to tumor treatment. This is because the high levels of growth factors, cytokines, and hormones in esophageal cancer cells can effectively promote cell proliferation, differentiation, metabolism, and autophagy (5, 6, 7). In addition, highly activated signal pathways mediated by growth factors and transmembrane receptors in tumor cells also greatly promote tumor growth and metastasis (8). In addition, based on a large sample size analysis of the genome, transcriptome and proteome studies have shown selective splicing and mutant genes; highly activated cell cycle–related pathways and receptor tyrosine signaling pathways play important roles in esophageal cancer (9, 10, 11, 12). These characteristics are important factors for determining the progression and therapeutic effects of esophageal cancer.

In order to cure esophageal cancer, the Food and Drug Administration (FDA) has approved several drugs for esophageal cancer treatment in the past decades (13, 14). However, drug discovery and development requires many stages, including discovery and development, preclinical research, clinical research, and FDA review, as well as FDA postmarket safety monitoring, which requires a large amount of money and time (15). Drug repositioning, the new use of old clinically approved drugs, can save time and cost with low risk (15, 16). Recent years have witnessed that several alternative drugs have been successfully applied in clinical trials, such as statins and metformin (17, 18). Sulconazole is listed as a broad-spectrum imidazole antifungal drug and has mainly been used to treat common skin fungal diseases in the UK since 1985 (19). Our previous study discovered that sulconazole was effective in inhibiting the growth of esophageal cancer cells both *in vitro* and *in vivo* (10). Here, we further demonstrate that sulconazole has significant inhibitory effects on a variety of tumor cells. It can not only inhibit the proliferation but also the migration of esophageal cancer cells. Mechanistically, sulconazole promoted PANoptosis by triggering oxidative stress and inhibiting glycolysis to increase radiosensitivity in esophageal cancer.

## Experimental Procedures

### Cell Lines and Reagents

Human cell lines (esophageal carcinoma cells KYSE30, KYSE150, and TE3; and esophageal epithelial cell line SHEE; liver carcinoma cells HepG2, Huh7, and normal Chang liver cells; gastric carcinoma cells SGC7901 and HGC27; lung carcinoma cells A549; breast carcinoma cells MDA-MB-453 and MCF7) were cultured in RPMI-1640 medium supplemented with 10% fetal bovine serum (FBS), penicillin (100 mg/ml), and streptomycin (100 mg/ml) and kept at 37 °C in a humidified atmosphere containing 5% CO_2_. All cell lines were tested for *mycoplasma* contamination. Sulconazole nitrate (HY-B1460A), Z-VAD-FMK (z-VAD) (HY-16658B), ferrostatin-1 (HY-100579), deferoxamine mesylate (HY-B0988), and necrostatin-1 (Nec) (HY-15760) were purchased from MedChemExpress and dissolved in dimethyl sulfoxide (DMSO) for storage, and the final concentration of DMSO used in all experiments did not exceed 0.1%.

### Cell Viability Assay (MTS Assay)

Cells were inoculated into 96-well plates at an initial density of 1 × 10^4^ cells per well. After cells became adherent, cells were refed with medium containing different concentrations of drugs and incubated for 24 h. Then, 20 μl MTS (G3518, Promega) was added to each well for 2 h. Finally, absorbance was measured at 492 nm using a Multiskan MK3 (Thermo Fisher Scientific). Cell viability curves were calculated and plotted with GraphPad Prism 8 software.

### Clonogenic Assay

KYSE30, KYSE150, and TE3 cells were inoculated into 12-well plates at 500 cells per well and cultured for 12 h until the cells adhered to the dish. Cells were treated with different concentrations of sulconazole for 24 h or different doses of ionizing radiation (IR) and then refed with fresh medium and cultured for 1 to 2 weeks. When there were at least 50 visible cell colonies in the culture plate, the cells were fixed with fixative (methanol:glacial acetic acid = 3:1), stained with 0.5% crystal violet, and then photographed with a Bio-Rad ChemiDoc MP. The number of clones was calculated with ImageJ (https://imagej.nih.gov/ij/) software and plotted with GraphPad Prism 8 (https://www.graphpad-prism.cn/) software.

### Transwell Assay

KYSE30 and KYSE150 cells were starved in serum-free medium for 12 h, then resuspended in serum-free medium, then 5 × 10^4^ cells were inoculated into the upper chamber of a transwell, and 500 μl medium containing 10% FBS was added to the lower chamber until cell had adhered. Cells were treated with serum-free medium containing DMSO or 20 μM sulconazole for 24 h. The cells that had migrated to the lower part of the chamber were stained with 0.5% crystal violet. After taking photos, ImageJ software was used to calculate the number of cells passing through the chamber, which was plotted with GraphPad Prism 8 software.

### Wound Healing Assay

KYSE30 and KYSE150 cells were inoculated into 6-well plates. When the cells reached confluence, a sterile tip was used to scratch the middle of each well. Cells were then washed with PBS and cultured in medium containing 2% FBS with DMSO or 20 μM sulconazole for 24 h. To ensure that measurements were made at the same locations, the locations were recorded using a calibration scale on the ix73 inverted microscope (Olympus). The same area was photographed at 0 and 24 h, and the wound healing distance was analyzed by ImageJ software and plotted using GraphPad Prism 8 software.

### RNA-Seq and Bioinformatic Analysis

KYSE30 and KYSE150 cells were treated with DMSO or sulconazole (50 μM). Total RNA was extracted with TRIzol (Life Technologies). BGI constructed the RNA library and analyzed the RNA sequences with a BGISEQ-500 system. Data were aligned to genomes using STAR (version 2.7.6a). The differentially expressed mRNAs were identified by DESeq2 (version 1.16.1). The mRNA differences with fold change >1.5 or <0.67 and *p*-value <0.05 were considered significantly upregulated or downregulated. These differential genes were entered into the Metascape database for KEGG and HALLMARK enrichment analysis (https://metascape.org/). The enriched signal pathways were mapped by R language and GraphPad Prism 8 software.

### Experimental Design and Statistical Rationale for Proteomics

Experiments were designed to investigate the mechanism by which sulconazole inhibits the progression of esophageal cancer cells and leads to cell death. The esophageal cancer cell line KYSE30 was treated with either DMSO (control group) or sulconazole (50 μM) (experimental group) in three biological replicates, then processed according to the filter aided sample preparation method. The TMT global proteomic sequencing method has been previously described by us (10). In short, tryptic peptides were desalinated and lyophilized with StageTips and then labeled with TMT-11plex (Pierce) according to the manufacturer's instructions. Finally, TMT MS experiments were performed on a nanoscale EASY-nLC 1200UHPLC system or nanoU3000UHPLC system (Thermo Fisher Scientific). The samples of the experimental group and the control group were compared by unpaired *t* test. Protein differences with fold change >1.3 or <0.77 and *p*-value <0.05 were considered significantly upregulated or downregulated. These differential proteins were entered into the Metascape database for KEGG and HALLMARK enrichment analysis (https://metascape.org/). The enriched signal pathways were mapped by R language and GraphPad Prism 8 software.

### MS Data Analysis and Database Search

Data were collected using Xcalibur software (Thermo Fisher Scientific, version 3.0) (https://thermo.flexnetoperations.com/control/thmo/index). Raw data were processed using Proteome Discoverer (https://thermo.flexnetoperations.com/control/thmo/index) (version 2.2), and MS/MS spectra were searched against the reviewed SwissProt human proteome database (20,311 entries). The release/download date was 29th August, 2020. All searches were carried out with a precursor mass tolerance of 20 ppm, fragment mass tolerance of 0.02 Da, oxidation (Met) (+15.9949 Da), TMT6plex (Lys) (229.163 Da), and acetylation (protein N-terminus) (+42.0106 Da) as variable modifications, carbamidomethylation (Cys) (+57.0215 Da), TMT6plex (N-terminal) (229.163 Da) as fixed modification, and three trypsin missed cleavages allowed. Only peptides of at least six amino acids in length were considered. Peptide and protein identifications were filtered by Proteome Discoverer to control a false discovery rate < 1%. At least one unique peptide was required for protein identification.

### Cell Death Assay

KYSE30 and KYSE150 cells were seeded at a density to achieve 70% confluence into 12-well plates. After cell adhesion, cells were treated with different concentrations of drugs for 12 h. To measure cell death, Annexin V/PI (C1062, Beyotime) was used for the detection of cell apoptosis or necroptosis. In addition, the cells were stained with 5 μg/ml propidium iodide (PI), and the percentage of the PI-positive cells was analyzed using a BD Accuri C6 flow cytometer. There were 1 × 10^4^ cells counted per sample. PI-positive cells may have undergone apoptosis, pyroptosis, necroptosis, or ferroptosis. For live cell imaging, 100 nM SYTOX Green (S7020, Thermo Fisher Scientific) was added for 20 min, then photographed using a Lionheart FX (BioTek). The resulting images were analyzed by ImageJ software, and the percentage of green-positive cells in each image was calculated.

### Glucose Uptake Assay

Methods for measuring glycolytic metabolic alterations of cellular metabolism have been described in our previous study (20). In short, 1 × 10^4^ KYSE30 and KYSE150 cells were inoculated into 96-well plates and treated with sulconazole (30 μM and 50 μM) for 24 h. Then, glucose uptake and lactate production were measured with a Glucose Uptake-Glo Assay kit (Promega).

### RNA Extraction and Quantitative RT-PCR

KYSE30 cells were treated with DMSO and sulconazole (30 μM), and KYSE150 cells were treated with DMSO and sulconazole (50 μM) for 24 h. Total RNA was extracted with TRIzol (15596018, Life Technologies). Then, reverse transcription to complementary DNA was performed using HiScript III RT SuperMix for qPCR (+gDNA wiper) (R323-01, Vazyme). Quantitative RT-PCR was performed using ChamQ Universal SYBR qPCR Master Mix (Q711-02, Vazyme) and an Applied Biosystems 7500/7500 Fast Real-Time PCR System (Thermo Fisher Scientific). The primers are listed in the supplemental Table S1.

### Western Blotting

Protein extraction and Western blotting were performed as described previously (21). Briefly, extracted proteins were separated by 8% - 15% SDS-PAGE. After the proteins were electrophoretically transferred to polyvinylidene fluoride (IPVH00010, Millipore) membrane, nonspecific binding was blocked by incubation with 5% skim milk, then membranes were incubated with the following primary antibodies: anti-PARP (CST, 9542S, 1:1000), anti-BCL2 (Proteintech, 12789-1-AP, 1:1000), anti-BAX (Proteintech, 50599-2-Ig, 1:1000), anti-caspase3 (CST, 14220, 1:1000), anti-cleaved caspase3 (CST, 9661S, 1:1000), anti-GSDME (Abcam, ab215191, 1:1000), anti-GSDMD (Proteintech, 20770-1-AP, 1:1000), anti-MLKL (Proteintech, 21066-1-AP, 1:1000), anti-pMLKL (Abcam, ab196436, 1:1000), anti-RIPK1 (CST, 3493T, 1:1000), anti-HK Ⅰ (CST, 8337T, 1:1000), anti-HK Ⅱ (CST, 8337T, 1:1000), anti-PFKP (CST, 8337T, 1:1000), anti-PKM1/2 (CST, 8337T, 1:1000), anti-PKM2 (CST, 8337T, 1:1000), anti-LDHA (CST, 8337T, 1:1000), anti-PDH (CST, 8337T, 1:1000), anti-AKT (CST, 4685S, 1:1000), anti-pAKT (CST, 4060S, 1:1000), anti-MEK1 (SB, 101351-T38, 1:1000), anti-pMEK (CST, 9121S, 1:1000), anti-ERK (CST, 4695S, 1:1000), anti-pERK (CST, 4370T, 1:1000), anti-STAT3 (CST, 9139S, 1:1000), and anti-pSTAT3 (CST, 9145S, 1:1000). Membranes were then washed and incubated with the appropriate horseradish peroxidase–conjugated secondary antibodies: anti-rabbit IgG (CST, 7074S, 1:2000) and m-IgGκ BP-HRP (Santa Cruz, sc-516102, 1:4000). A ChemiDoc MP Imaging System was used to visualize the proteins (Bio-Rad).

### LDH Release Assay

Cells were seeded in 96-well plates at 1 × 10^4^ cells per well. After 12 h, different concentrations of drugs were added for an additional 12 h. Then, the supernatants of the cultures were transferred to new wells, and lactate dehydrogenase (LDH) was measured with a CytoTox 96 Non-Radioactive Cytotoxicity Assay kit (G1780, Promega). Finally, absorbance was measured at 492 nm with a Thermo Multiskan MK3 (Thermo Fisher Scientific). The LDH release ratio was calculated by the following equation: (LDH _sample_ - LDH _background_ - LDH _untreated_)/(LDH _maximum_ - LDH _background_) × 100%.

### ROS Production, Mitochondrial Membrane Potential, and Lipid Peroxidation Assays

KYSE30 and KYSE150 cells were inoculated into 12-well plates. After cell attachment to the dish, cells were treated with DMSO and sulconazole (50 μM) for 24 h to measure reactive oxygen species (ROS) production and mitochondrial membrane potential or 12 h to measure lipid peroxidation. Then, cells were harvested and washed with Hank’s Balanced Salt Solution and incubated cells with 5 μM MitoSOX (M36008, Thermo Fisher Scientific), 100 nM TMRE (HY-D0985A, MedChemExpress), and 5 μM BODIPY 581/591 C11 (D3861, Thermo Fisher Scientific) for 30 min at 37 °C, protected from light. Then, cells were washed twice with Hank’s Balanced Salt Solution and analyzed using a BD Accuri C6 flow cytometer.

### Transmission Electron Microscopy

Transmission electron microscopy (TEM) analysis was performed with a JEM-F200 transmission microscope (JEOL). Samples were fixed with a solution containing 2.5% glutaraldehyde for more than 2 h at 4 °C, then washed in PBS buffer three times at 37 °C for 40s each time. Samples were treated with 0.1% Millipore-filtered cacodylate-buffered tannic acid, postfixed with 1% buffered osmium, and stained *en bloc* with 1% Millipore-filtered uranyl acetate. Samples were dehydrated in 50%, 70%, and 90% ethanol, respectively, and finally washed with anhydrous ethanol three times, then infiltrated and embedded in LX-112 medium. Medium was allowed to polymerize at 40 °C for 30 min, 60 °C for 10 min, 70 °C for 10 min, and 80 °C for 20 min, then samples were cut into 60 to 80 nm thick sections on an ultrathin sectioning machine, stained with uranyl acetate for 30 min and lead citrate for 5 min, and then examined with a JEM-F200 transmission electron microscope at an accelerating voltage of 80 kV. Digital images were obtained using an AMT Imaging System (Advanced Microscopy Techniques Corp).

### Statistical Analyses

Statistical analyses were performed using GraphPad Prism 8. All experimental results are described by means ± SD. Independent groups of samples were evaluated using an unpaired two-sided Student’s *t* test. For comparisons between multiple groups, a two-way ANOVA was used. All specific statistical details are displayed in the figure captions and source data. All statistically relevant experiments were performed with at least three biological replicates.

## Results

### Sulconazole has a Significant Anticancer Effect

**Fig. 1:**
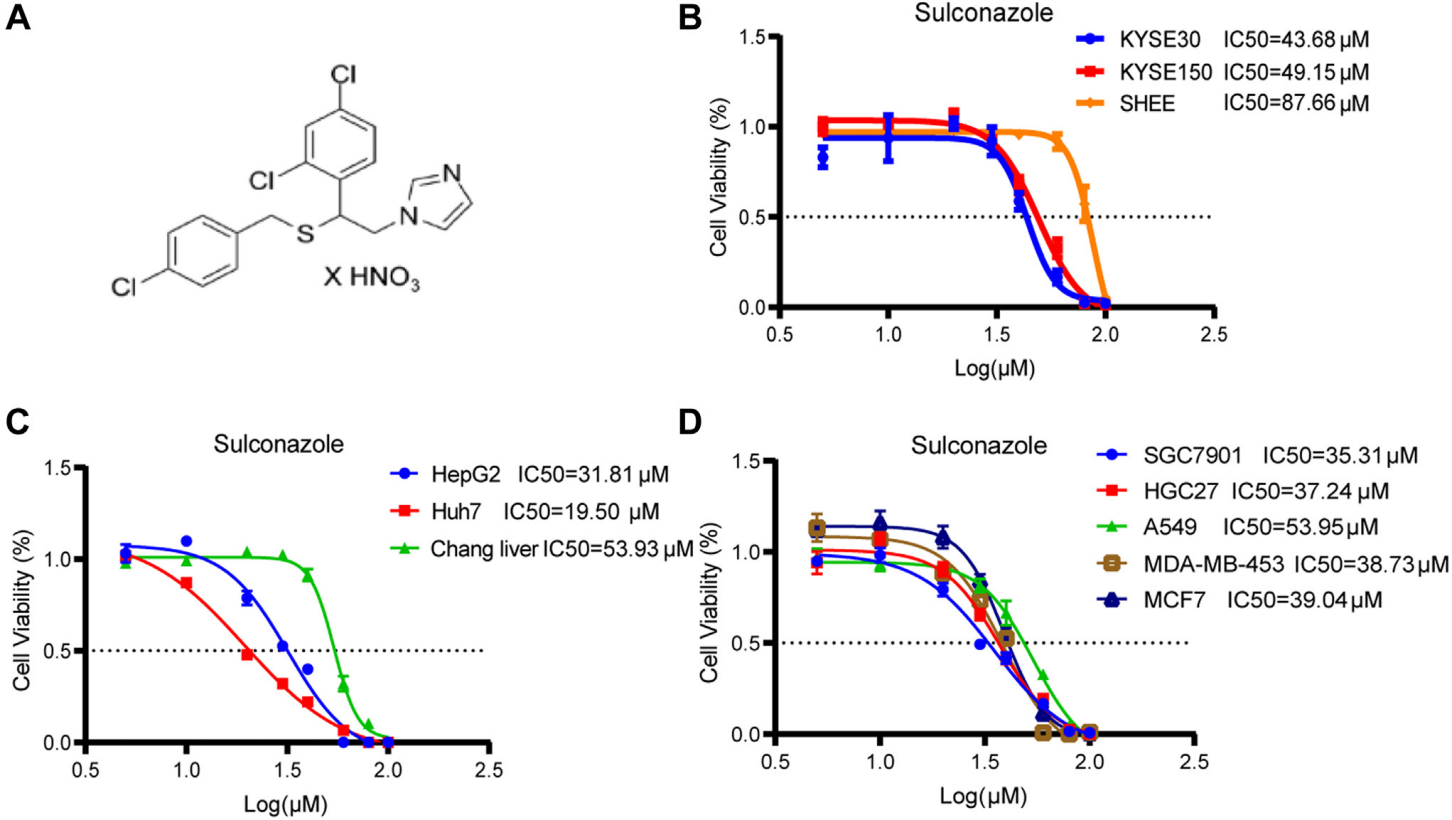
**Sulconazole inhibits the viability of various cancer cell lines.***A*, molecular structure of sulconazole. *B*–*D*, viability of esophageal cancer cell lines (KYSE30 and KYSE150) and an esophageal epithelial cell line (SHEE) (*B*), liver cancer cell lines (HepG2 and Huh7) and a normal liver cell line (Chang liver) (*C*), gastric cancer cell lines (SGC7901 and HGC27), lung cancer cell line (A549), and breast cancer cell lines (MDA-MB-543 and MCF7) (D) were assessed after treatment with sulconazole at concentrations as indicated for 24 h. The data are representative of three independent experiments and presented as the mean ± SD.

We first evaluated the anticancer effect of sulconazole through cell function experiments *in vitro*. Cell viability assays showed that the IC50 (half maximal inhibitory concentration) of sulconazole on the esophageal cancer KYSE30 and KYSE150 cell lines was 43.68 μM and 49.15 μM, respectively, and on the SHEE esophageal epithelial cell line was 87.66 μM (Fig. 1*B*). The IC50 for HepG2 and Huh7 liver cancer cells was 31.81 μM and 19.50 μM, respectively, and 53.93 μM for the normal Chang liver cell line (Fig. 1*C*). In addition, it also had significant inhibitory effect on gastric cancer (SGC7901, IC50 = 35.31 μM and HGC27, IC50 = 37.24 μM), lung cancer (A549, IC50 = 53.59 μM), and breast cancer (MDA-MB-453, IC50 = 38.73 μM and MCE7, IC50 = 39.04 μM) cell lines (Fig. 1*D*). These results suggest that sulconazole has a broad-spectrum anticancer effect.

### Sulconazole Inhibits the Proliferation and Migration of Esophageal Cancer Cell Lines

**Fig. 2:**
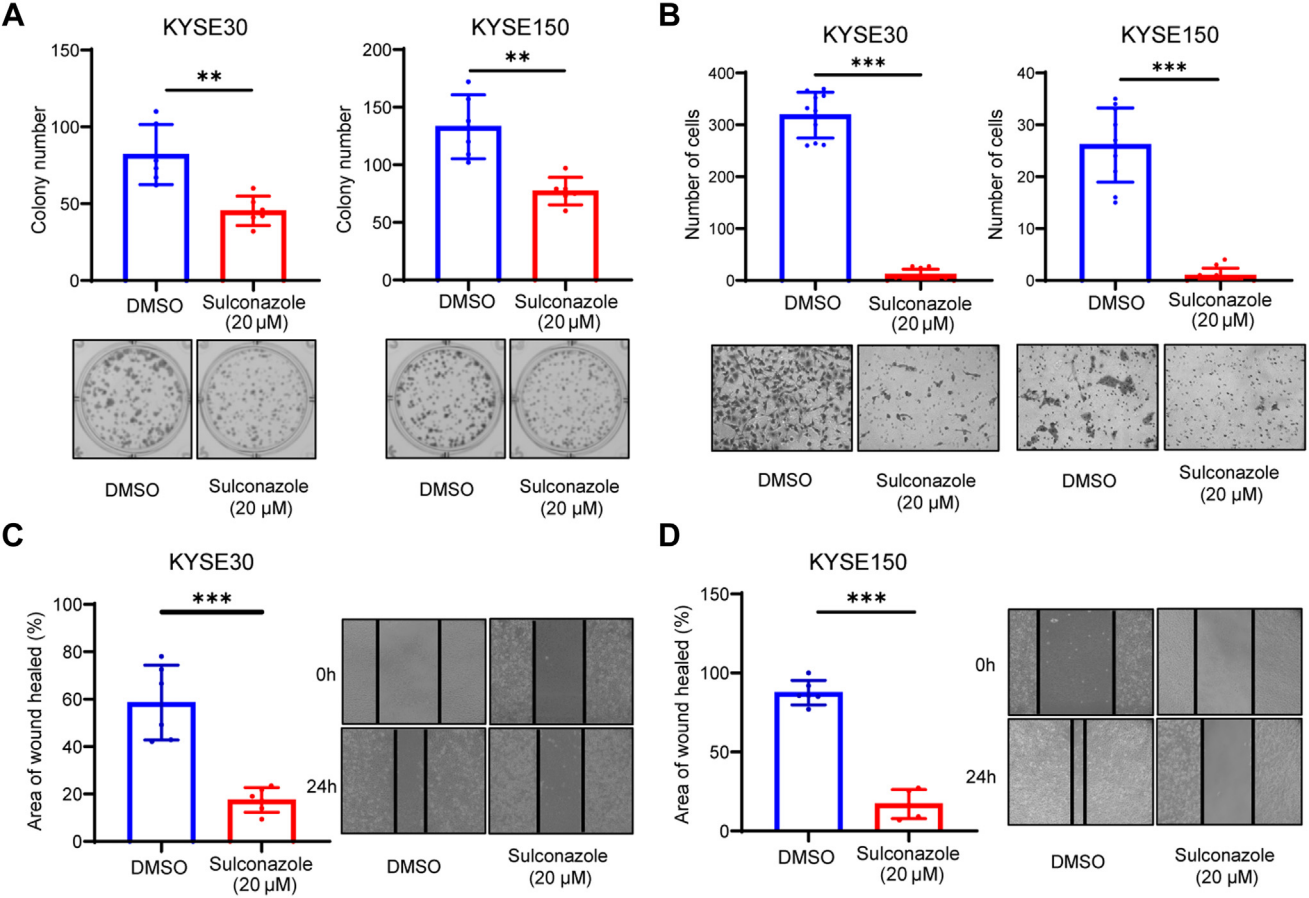
**Sulconazole inhibits the proliferation and migration of esophageal cancer cell lines.***A*, cell clonogenic assay was used to verify the inhibition of proliferation of KYSE30 (*left panel*) and KYSE150 (*right panel*) cells after sulconazole treatment for 24 h. *B*, transwell assay was used to verify the inhibitory effect of sulconazole on migration of KYSE30 (*left panel*) and KYSE150 (*right* panel) cells after sulconazole treatment for 24 h. The number of cells was counted in random fields. *C* and *D*, migration of KYSE30 and KYSE150 cells was detected by wound healing assay after sulconazole treatment for 24 h. Diagrams (*left panel*) were used for quantitative analyses of migration distance. All data are representative of three independent experiments. *p*-values were calculated by unpaired two-sided Student’s *t* tests. ∗∗*p* < 0.01, ∗∗∗*p* < 0.001.

The cell clonogenic assay results showed that 20 μM sulconazole could effectively inhibit colony formation of esophageal cancer cells (Fig. 2*A*). Transwell assays showed that sulconazole markedly reduced the number of cells invading through the chamber (Fig. 2*B*). In a wound healing assay, sulconazole inhibited the wound healing rate of KYSE30 and KYSE150 cells (Fig. 2, *C* and *D*). In conclusion, our results suggest that sulconazole effectively inhibits the progression of esophageal cancer cells *in vitro*.

### Dysregulated Genes and Proteins Based on Transcriptomics and Proteomics Following Sulconazole Treatment

**Fig. 3:**
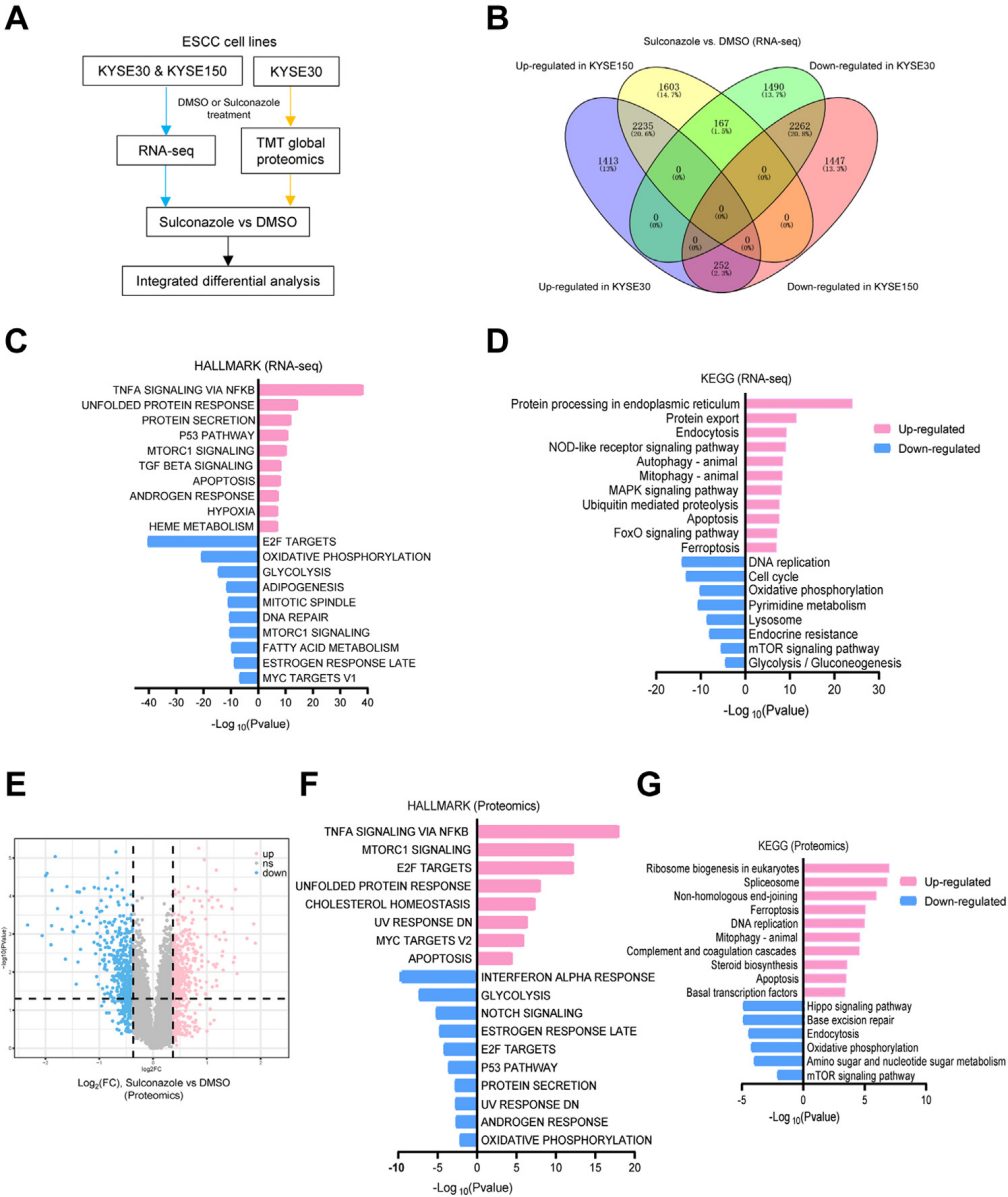
**Dysregulated proteins and pathways in esophageal cancer cells treated with sulconazole were analyzed by transcriptomics and proteomics.***A*, flowchart of the experimental process of transcriptomic sequencing and proteomic sequencing. *B*, Venn diagram of 1.5-fold change upregulated and downregulated genes from transcriptomic sequencing. *C*, HALLMARK enrichment analysis of dysregulated genes. *D*, KEGG enrichment analysis of dysregulated genes. *E*, volcano plot of 1.3-fold change upregulated and downregulated proteins from proteomic sequencing. *F*, KEGG enrichment analysis of dysregulated proteins. *G*, HALLMARK enrichment analysis of dysregulated proteins.

To investigate the underlying molecular mechanism(s) of sulconazole-mediated inhibition of esophageal cancer cells, RNA and protein of esophageal cancer cells treated with sulconazole were extracted and the transcriptomes (KYSE30 and KYSE150) and proteome (KYSE30) were sequenced (Fig. 3*A*). After sulconazole treatment, a large number of genes were significantly upregulated and downregulated (FC > 1.5 or <0.67, *p* < 0.05). There were 2235 genes upregulated, and 2262 genes downregulated in both KYSE30 and KYSE150 cell lines (Fig. 2*B*). KEGG and HALLMARK analysis of differentially expressed genes showed that the upregulated genes resulting from sulconazole treatment were mostly enriched in apoptosis, ferroptosis, autophagy, mitophagy, and related signal pathways. Downregulated genes were focused on DNA replication/repair, cell cycle, glycolysis, and oxidative phosphorylation (Fig. 2, *C* and *D*). Proteomic sequencing showed 419 proteins were upregulated and 434 proteins were downregulated (FC > 1.3 or <0.77, *p* < 0.05) (Fig. 2*E*). Similarly, proteome results were also enriched in autophagy, apoptosis, and ferroptosis, in addition to downregulation of glycolysis and oxidative phosphorylation (Fig. 2, *F* and *G*). These results indicate that sulconazole suppresses esophageal cancer progression by inhibiting glycolysis and promoting multiple types of programmed cell death (PCD).

### Sulconazole Induces PANoptosis of Esophageal Cancer Cells

**Fig. 4:**
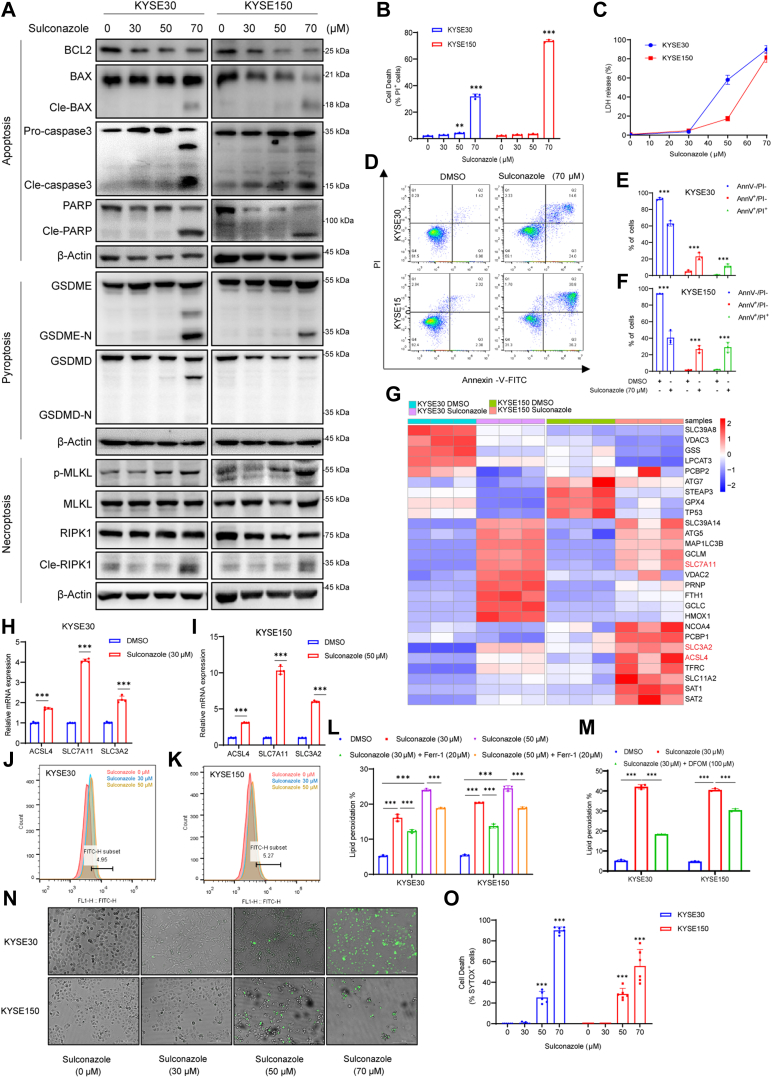
**Sulconazole induces PANoptosis of esophageal cancer cells.***A*, Western blot analyses for the expression of BCL2, BAX, cleaved BAX, caspase3, cleaved caspase3, PARP, cleaved PARP, GSDME, GSDME-N, GSDMD, GSDMD-N, p-MLKL, MLKL, RIPK1, cleaved RIPK1, and β-Actin after sulconazole treatment for 12 h. *B*–*D*, flow cytometry (*B*) and quantification analysis (*C* and *D*) with Annexin V/PI staining evaluating the percentages of live cells in Q4 (Annexin V^-^/PI^-^), early apoptotic cells in Q3 (Annexin V^+^/PI^-^), and late apoptotic cells and necrotic cells in Q2 (Annexin V^+^/PI^+^) among KYSE30 and KYSE150 treated with DMSO or sulconazole for 12 h. *E*, cell death in KYSE30 and KYSE150 cells treated with sulconazole for 12 h were detected by flow cytometry, and the PI-positive cells were calculated and shown in the diagrams. *F*, LDH release of KYSE30 and KYSE150 cells after sulconazole treatment for 12 h. *G*, heat map of ferroptosis-related genes that were differentially expressed in KYSE30 and KYSE150 cells with or without sulconazole treatment. *H* and *I*, the mRNA expression of ferroptosis-related genes by quantitative RT-PCR after sulconazole treatment for 24 h *J*–*M*, measurement of lipid peroxidation after sulconazole Ferr-1 (20 μM) and DFOM (100 μM) treatment for 12 h. Bar graph showing relative levels of lipid peroxidation by C11-BODIPY staining in KYSE30 and KYSE150 cells (*L* and *M*). *N*, representative images of cell death in KYSE30 and KYSE150 cells after sulconazole treatment for 12 h. *O*, diagrams were used for quantitative analyses of green dead cells (SYTOX Green-positive) in (*N*). The data in (*B*–*O*) are representative of three independent experiments and presented as the mean ± SD. *p*-values were calculated by two-way ANOVA. ∗∗*p* < 0.01, ∗∗∗*p* < 0.001. DFOM, deferoxamine mesylate; Ferr-1, ferrostatin-1; LDH, lactate dehydrogenase; MLKL, mixed lineage kinase-like domain; PI, propidium iodide.

PANoptosis usually considered regulated by PANoptosome complex is an inflammatory PCD. In addition, the most important point is that PANoptosis cannot be characterized by apoptosis, pyroptosis, necroptosis, and ferroptosis alone (22, 23). In addition, studies have shown that chemotherapeutic drugs can also induce noninflammasome-dependent PANoptosis (24). Based on the results of the above analysis, we explored whether sulconazole could induce PANoptosis. First, we found that esophageal cancer cells treated with sulconazole resulted in time-dependent and dose-dependent cell death, including PI-positive cells that are indicative of cellular apoptosis, necroptosis, pyroptosis, or ferroptosis (Figs. 4*B* and S1, *A* and *C*). Meanwhile, we also collected cell lysates for Western blot detection and found that poly ADP-ribose polymerase (PARP) and cleaved PARP also changed accordingly (Figs. 4*A* and S1, *B* and *D*), which indicates apoptosis. Next, in order to explore how sulconazole triggers apoptosis, we treated esophageal cancer cells in a dose-dependent manner at the same time (12 h) and used Western blotting to detect apoptosis-related upstream signal pathways. We found that BCL2 decreased and cleaved BAX and cleaved caspase3 increased after sulconazole treatment (Fig. 4*A*). It has been reported that during chemotherapy, pyroptosis can occur with apoptosis and depends on the activity of caspase3 (25), so we tested the pyroptosis effector proteins GSDME and GSDMD and found that the cleaved GSDME, but not cleaved GSDMD, was increased. A simultaneous LDH assay showed that the release of LDH also increased (Fig. 4, *A* and *C*). Further, after combination of sulconazole and caspase inhibitor z-VAD (Z-VAD-FMK), cleavage of both PARP and GSDME was obviously inhibited and the percentage of dead cells was also reduced (supplemental Fig. S1, *E* and *F*), The above results combined show that sulconazole can induce apoptosis and pyroptosis *via* a BCL2–Bax–caspase3 axis. Flow cytometry analysis after treatment of cells with sulconazole and staining with Annexin V/PI showed that the percentages of live cells in Q4 (Annexin V^-^/PI^-^) decreased, whereas early apoptotic cells in Q3 (Annexin-V^+^/PI^-^) and late apoptotic cells and necrotic cells in Q2 (Annexin V^+^/PI^+^) increased (Fig. 4, *D*–*F*), suggesting that cell necroptosis also occurs. We found that following sulconazole treatment, cells also showed robust phosphorylation of pseudokinase mixed lineage kinase-like domain (MLKL), an inducer of necroptosis (26). Accordingly, we noted enhanced levels of cleaved RIPK1 (Fig. 4*A*), an upstream molecule that induces the phosphorylation of MLKL and participates in the crosstalk between apoptosis and necroptosis (27). Consistent with this, necrostatin-1 (Nec), an inhibitor of RIPK1, could partially rescue the cell death caused by sulconazole. This indicates that necroptosis also occurs. Moreover, heat map results showed that the expression of a large number of ferroptosis-related genes changed (Fig. 4*G*); we also found that the mRNA expression of ACSL4, an upstream molecule of ferroptosis, and SLC7A11 and SLC3A2, the ferroptosis feedback factors, increased significantly after sulconazole treatment, based on quantitative RT-PCR (Fig. 4, *H* and *I*). These results suggest that ferroptosis also occurred. Ferroptosis is an iron-dependent regulatory cell death form caused by excessive lipid peroxidation (28). Consequently, the level of lipid peroxidation significantly increased with sulconazole in a dose-dependent manner and could be markedly inhibited by the lipid peroxidation inhibitor ferrostatin-1 and iron chelator deferoxamine mesylate (Fig. 4, *J*–*M*). These results indicate that sulconazole induced ferroptosis in tumor cells. Although a variety of death inhibitors can partly rescue sulconazole-induced cell death, none of the inhibitors could completely prevent cell death (supplemental Fig. S1*F*). Finally, we used a commonly used PANoptosis probe to stain the cells and showed that SYTOX-positive cells increased with the increase of drug concentration (Fig. 4, *H* and *I*). Taken together, these data demonstrate that sulconazole induces PANoptosis in esophageal cancer cells.

### Sulconazole Triggers Mitochondrial Oxidative Stress and Inhibits Glycolysis *via* Downregulating HK

**Fig. 5:**
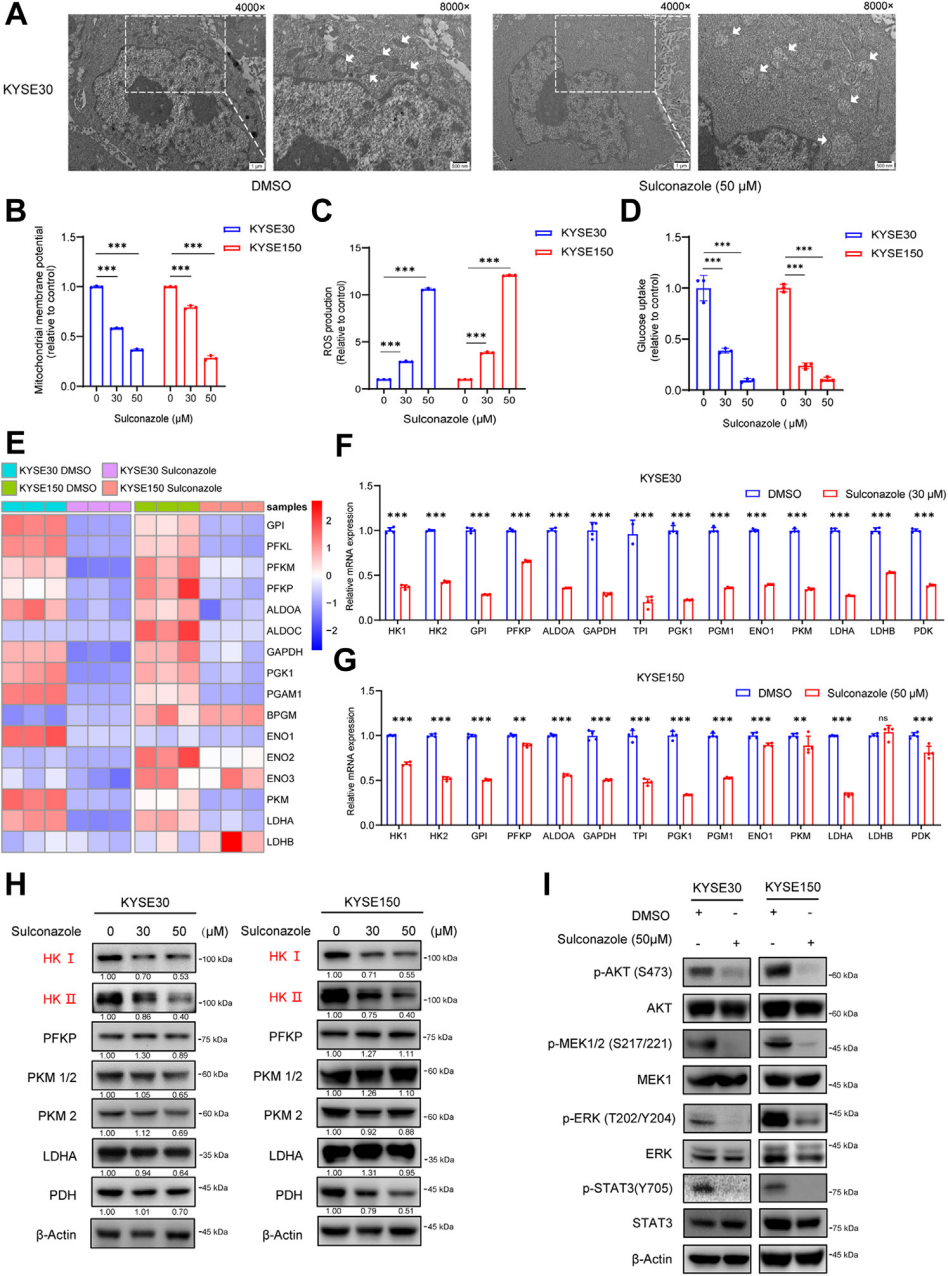
**Sulconazole triggers mitochondrial oxidative stress and inhibits glycolysis.***A*, transmission electron microscopy (TEM) images of KYSE30 cells subjected to the indicated treatments for 24 h. *White* arrows indicate mitochondria. Scale bars represent left, 1 μm; right, 500 nm. *B*; PI, propidium iodide*D*, mitochondrial membrane potential analysis (*B*), ROS level analysis (*C*), and glucose uptake analysis (*D*) in KYSE30 and KYSE150 cells after sulconazole treatment for 24 h. *E*, heat map of 19 glycolysis-related enzymes that were differentially expressed in KYSE30 and KYSE150 cells with or without sulconazole treatment. *F* and *G*, the mRNA expression of glycolysis pathway enzymes by quantitative RT-PCR in KYSE30 (*F*) and KYSE150 (*G*) cells. *H*, Western blot analyses of glycolysis-related enzymes after sulconazole treatment for 24 h in KYSE30 (*left* panel) and KYSE150 (*right panel*) cells. Quantification of the blots is shown below. *I*, Western blot analyses for the expression of p-AKT, AKT, p-MEK, MEK, p-ERK, ERK, p-STAT3, and STAT3 after sulconazole treatment for 24h in KYSE30 (*left panel*) and KYSE150 (*right* panel) cells. The data in (*B*–*D*, *F* and *G*) are representative of three independent experiments and presented as the mean ± SD. *p*-values were calculated by two-way ANOVA. ∗∗*p* < 0.01, ∗∗∗*p* < 0.001. ROS, reactive oxygen species.

Oxidative phosphorylation and glycolysis are important pathways for energy production in cells (29), and mitochondria play an important role in cell energy metabolism. Studies have shown that mitochondrial dysfunction can activate the BCL2–Bax–Caspase3 pathway and release cytochrome C and ROS to promote various types of PCD (30). TEM revealed that esophageal cancer cells (KYSE30) treated with sulconazole (50 μM) exhibited shrunken mitochondria with increased membrane density and chromatin margination in the nucleus, which is a characteristic morphologic feature of early cell apoptosis (Fig. 5*A*). We also detected the changes in mitochondrial membrane potential and the production of ROS. The results showed that with increasing of drug concentration, the mitochondrial membrane potential gradually decreased and intracellular ROS production increased (Fig. 5, *B* and *C*). Meanwhile, glucose uptake was significantly inhibited under sulconazole treatment (Fig. 5*D*). Heat map analysis from transcriptomics showed that many glycolysis-related enzyme genes were dysregulated (Fig. 5*E*), as was the proteome (data not shown). Next, the quantitative RT-PCR and Western blotting were employed to validate the expression levels of these genes. The results showed that many glycolysis-related enzymes decreased at the transcriptional level (Fig. 5, *F* and *G*), but at the protein level, only HK1 and HK2 were significantly downregulated (Fig. 5*H*). AKT, MEK/ERK, and STAT3 pathways were effectively inhibited after sulconazole treatment (Fig. 5*I*). These results indicate that sulconazole inhibits glycolysis and causes mitochondrial oxidative stress through multiple signal pathways, such as the AKT, MEK/ERK, and STAT3 pathways, resulting in the PANoptosis of tumor cells.

### Sulconazole Increases Radiosensitivity of Esophageal Cancer Cells

**Fig. 6:**
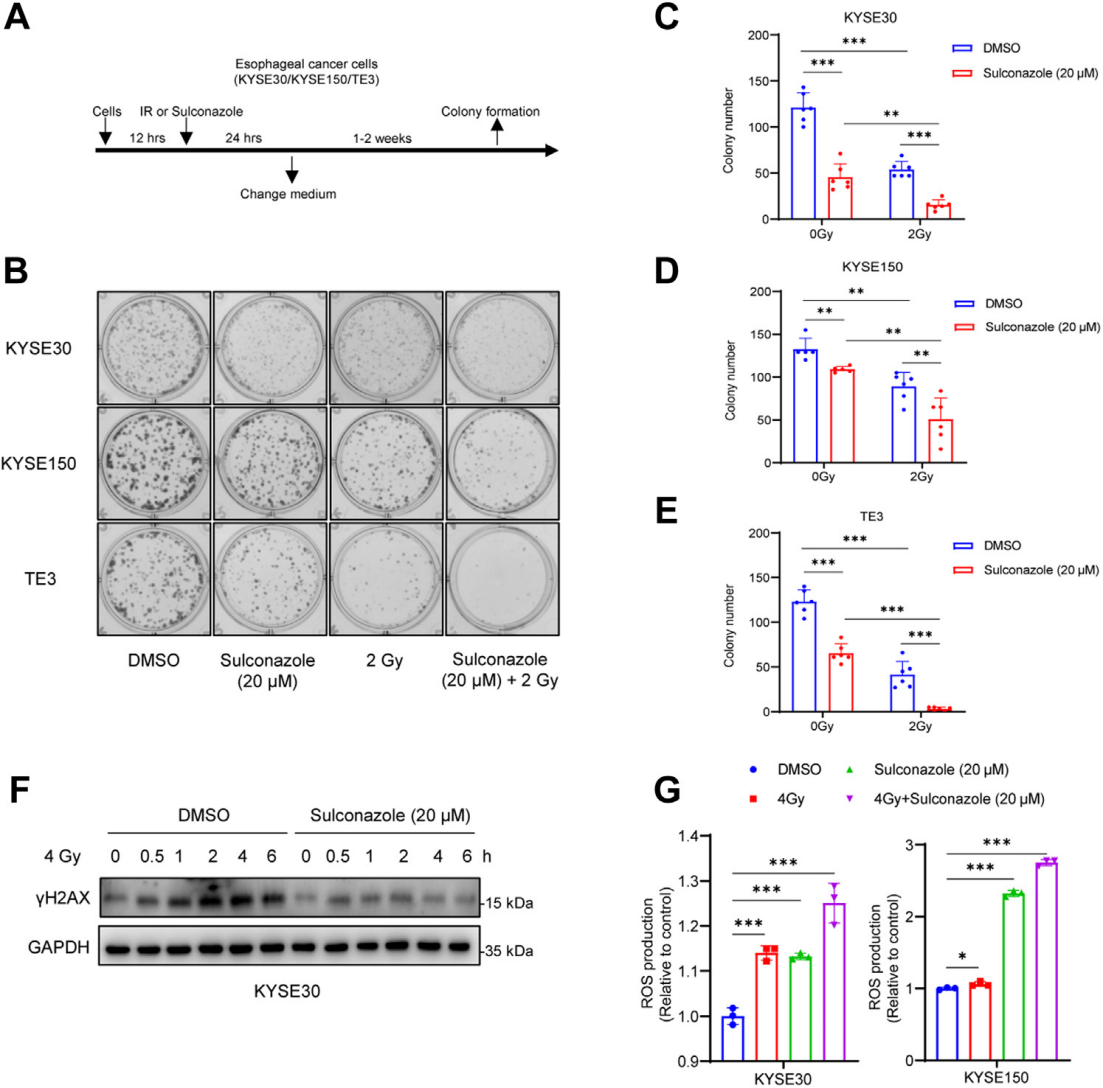
**Sulconazole increases radiosensitivity of esophageal cancer cells.***A*, flowchart of the clonogenic assay under sulconazole/IR chemoradiotherapy treatment. *B*, typical diagrams of radiation survival of KYSE30, KYSE150, and TE3 cells. *C*, *D* and *E*, statistical histogram of numbers of colonies formed. *F*, KYSE30 was treated with DMSO or sulconazole (20 μM) for 24 h, and γH2AX was detected at different time points after 4 Gy irradiation. *G*, ROS levels were detected after treatment with DMSO (control), sulconazole (20 μM), 4 Gy, or sulconazole (20 μM) combined with 4 Gy for 24 h in KYSE30 and KYSE150 cells. The data in (*C*–*E*, *G* and *H*) are representative of three independent experiments and presented as the mean ± SD. *p*-values were calculated by two-way ANOVA (*C*–*E*) and unpaired two-sided Student’s *t* test (*G* and *H*). ∗*p* < 0.05, ∗∗*p* < 0.01, ∗∗∗*p* < 0.001. IR, ionizing radiation; ROS, reactive oxygen species.

Radiotherapy is the primary treatment for esophageal cancer. We explored the effect of low-dose sulconazole on radiosensitivity of esophageal cancer cells through a radiation survival assay (Fig. 6*A*). Three esophageal cancer cell lines, KYSE30, KYSE150, and TE3, were treated with DMSO (control), sulconazole (20 μM), 2 Gy, and sulconazole (20 μM) combined with 2 Gy. The results showed that low-dose sulconazole could significantly increase the radiosensitivity of esophageal cancer cells (Fig. 6, *B*–*E*). Next, we detected γH2AX, in DMSO- and sulconazole-treated cells, at different times after 4 Gy irradiation. The results substantiated that sulconazole inhibits the response to DNA damage and repair in tumor cells (Fig. 6*F*). The combination of sulconazole and irradiation also elevated intracellular ROS (Fig. 6, *G* and *H*), which indicates that sulconazole can lead to the radiosensitivity of tumor cells by promoting oxidative stress.

## Discussion

Sulconazole is a conazole antifungal drug that has been used to treat skin fungal infection and other diseases since 1985. Because the third nitrogen atom on the imidazole group in the sulconazole molecule can selectively bind and inhibit the activity of sterol 14α-demethylase (CYP51) on the fungal cell membrane, the fungus cannot synthesize ergosterol, which is a key component of the cell membrane (31). Moreover, CYP51, is an important prognostic marker in cancer and is abnormal in many cancers, such as colorectal cancer (32), breast cancer (33), and ovarian cancer (34). Rapidly proliferating tumor cells synthesize more cholesterol to form cell membranes. The intracellular pathway involving CYP51 is fully committed to sterol production, which makes it possible to become a more selective drug target (35). Studies have demonstrated that sulconazole can inhibit breast cancer stem cell formation through NF-κB/IL8 signaling (36), but there is no evidence for its use in the treatment of esophageal cancer. Our previous results showed that sulconazole can significantly inhibit the proliferation of tumor cells *in vivo* and *in vitro* (10). In this study, we examined that sulconazole has broad spectrum anticancer activity with less harmful effects on normal cells. Further, sulconazole can also inhibit the migration of tumor cells and ultimately lead to PANoptosis.

PANoptosis is activated by components of pyroptosis, apoptosis, and necroptosis. It cannot be grouped into any of the three types of cell death (37). Here, we found that sulconazole induces apoptosis, pyroptosis, necroptosis, and ferroptosis simultaneously (Fig. 4). When oxidative stress occurs in mitochondria, a large amount of ROS will be produced and cytochrome C will be released. The abnormal expression of BCL2 family proteins on mitochondria triggers the activation of caspase and cleavage of PARP, leading to apoptosis (38, 39, 40, 41, 42). Caspase3 specifically cleaves GSDME to release its N-terminus to perforate the cell membrane, resulting in the release of intracellular lysates and leading to pyroptosis (25). Cell necrosis is initiated by the tumor necrosis factor receptor and Toll-like receptor families, as well as death signals through two protein kinases RIP1 and RIP3 that interact with receptor proteins, activating its specific substrate protein MLKL, thus leading to necroptosis (43). These are all related to mitochondrial dysfunction. It is interesting that sulconazole can also induce ferroptosis. Ferroptosis is a PCD caused by iron-dependent lipid peroxidation. An important indicator of ferroptosis is the decrease or disappearance of cristae. The rupture and collapse of the outer membrane of mitochondria and the large production of intracellular ROS are also closely related to the dysfunction of mitochondria (30, 44, 45, 46). Through TEM, we did see that tumor cells showed altered mitochondrial morphology and pre-apoptotic characteristics after sulconazole treatment (Fig. 5*A*). Therefore, based on the above, we speculate that sulconazole may cause cell PANoptosis through affecting mitochondrial functions.

As an important energy producing organelle, mitochondria play a decisive role in regulating cell fate. Glycolysis is the most important glucose metabolism pathway in tumor cells. Many of its intermediates provide important raw materials for normal metabolism, such as for the tricarboxylic acid cycle and oxidative phosphorylation in mitochondria. Studies have proven that many intermediate glycolytic enzymes can directly regulate the function of mitochondria. For example, PGK1 is able to translocate to mitochondria under hypoxia, thereby affecting the pyruvate metabolic pathway (47). Furthermore, not only is HK2 the rate-limiting enzyme of glycolysis, under hypoxia, its expression is also elevated and localized in mitochondria to inhibit ROS production, upregulating mitochondrial membrane potential and thus inhibiting cell death (48, 49). HK2 can also bind to mitochondrial membrane protein VDAC1, which is crucial to the carcinogenic effect of HK by promoting mitochondrial function and inhibiting apoptosis (50, 51). In addition, some studies have reported that HK1 on mitochondria can also inhibit apoptosis (52). Based on these studies, we suggest that sulconazole triggers mitochondrial dysfunction and inhibits glycolysis by downregulating HK expression (Fig. 5). Moreover, the high activation of PI3K–AKT–mTOR pathway has been used as an important therapeutic target in a variety of cancers (53, 54, 55). Activation of the MEK–ERK signaling pathway can promote the proliferation, differentiation, cell cycle, and drug resistance of tumor cells (56). As an important signal transducer and activator of transcription in cells, STAT3 is abnormally activated in a variety of cancers and promotes the progression of tumor cells and is also an important therapeutic target (57, 58, 59). Therefore, the inhibition of these pathways also plays an important role in the molecular mechanism of sulconazole anticancer function.

Radiotherapy and chemotherapy are commonly used as tumor treatment methods at present. The combination of radiotherapy and chemotherapy can reduce their dosage, toxicity, and side effects alone. We tried to explore whether sulconazole could be combined with radiotherapy to produce a better anticancer effect. The experimental results showed that the combined use of 20 μM sulconazole and 2 Gy had a better anticancer effect than the single treatment group (Fig. 6, *B*–*E*). Further, we found that the cells lost response to irradiation after sulconazole treatment, and sulconazole combined with radiation could promote the occurrence of oxidative stress in mitochondria (Fig. 6, *F*, *G* and *H*). This indicates that sulconazole may increase the radiosensitivity of tumor cells by promoting oxidative stress. In conclusion, we show that sulconazole increases radiosensitivity by triggering oxidative stress and inducing PANoptosis, and these results can provide guidance for clinical application.

## Conclusions

In this study, we show that sulconazole has good anticancer effect, can inhibit proliferation and migration, and induces PANoptosis of esophageal cancer cells. Mechanistically, sulconazole triggers oxidative stress and inhibits glycolysis *via* downregulating HKs and inhibiting the PI3K–AKT, MEK–ERK, STAT3 pathways. Finally, sulconazole can increase the radiosensitivity of esophageal cancer cells. These results effectively reposition sulconazole as an anticancer candidate drug and provide sufficient experimental evidence for the clinical application of esophageal cancer.

## Data Availability

The mass spectrometry proteomics data have been deposited in the ProteomeXchange Consortium (http://proteomecentral.proteomexchange.org) *via* the iProX partner repository (60, 61) with the dataset identifier PXD041175.

## Supplemental data

This article contains supplemental data.

## Conflict of interest

The authors declare no conflicts of interest with the contents of this article.

## Supplemental Data

Supplementary Figure S1

Original Western Blots
