# Supplementary material for: Sulconazole Induces PANoptosis by Triggering Oxidative Stress and Inhibiting Glycolysis to Increase Radiosensitivity in Esophageal Cancer
Source: Mol Cell Proteomics. 2023 Apr 17;22(6):100551. doi: 10.1016/j.mcpro.2023.100551 (PMC10205543; doi:10.1016/j.mcpro.2023.100551)
Supplement: Supplemental Table S1 [file mmc1.docx]

**Sulconazole induces PANoptosis by triggering oxidative stress and inhibiting glycolysis to increase radiosensitivity in esophageal cancer**

Lu-Xin Liu ^1,2^, Jing-Hua Heng ^1,2^, Dan-Xia Deng ^1,2^, Hui Zhao ^2,3^, Zhen-Yuan Zheng ^1,2,3^, Lian-Di Liao ^1,2^, Wan Lin ^1,3^, Xiu-E Xu ^1,2^, En-Min Li ^2*^, and Li-Yan Xu ^1,2,3*^

**Supplementary Table Legend:**

**Supplementary Table S1. The primers for qualitative RT-PCR assay**

**Supplementary Table S2. Identified proteins of proteomic sequencing**

| **Supplementary Table S1. The primers for qualitative RT-PCR assay** | |
| --- | --- |
| **Primers** | **Sequences (5’- 3’)** |
| HK1-qF | CACATGGAGTCCGAGGTTTATG |
| HK1-qR | CGTGAATCCCACAGGTAACTTC |
| HK2-qF | GATTGCCTCGCATCTGCTTG |
| HK2-qR | GCTCCAAGCCCTTTCTCCAT |
| GPI-qF | CCGCGTCTGGTATGTCTCC |
| GPI-qR | CCTGGGTAGTAAAGGTCTTGGA |
| PFKP-qF | GACCTTCGTTCTGGAGGTGAT |
| PFKP-qR | CACGGTTCTCCGAGAGTTTG |
| ALDOA-qF | ATGCCCTACCAATATCCAGCA |
| ALDOA-qR | GCTCCCAGTGGACTCATCTG |
| GAPDH-qF | ACAACTTTGGTATCGTGGAAGG |
| GAPDH-qR | GCCATCACGCCACAGTTTC |
| TPI1-qF | TCGTTGGGGGAAACTGGAAG |
| TPI1-qR | CAGTAGGGGGAGCACAAACC |
| PGK1-qF | GACCTAATGTCCAAAGCTGAGAA |
| PGK1-qR | CAGCAGGTATGCCAGAAGCC |
| PGM1-qF | AGCATTCCGTATTTCCAGCAG |
| PGM1-qR | GCCAGTTGGGGTCTCATACAAA |
| ENO1-qF | TGGTGTCTATCGAAGATCCCTT |
| ENO1-qR | CCTTGGCGATCCTCTTTGG |
| PKM-qF | ATGTCGAAGCCCCATAGTGAA |
| PKM-qR | TGGGTGGTGAATCAATGTCCA |
| LDHA-qF | TTGACCTACGTGGCTTGGAAG |
| LDHA-qR | GGTAACGGAATCGGGCTGAAT |
| LDHB-qF | CCTCAGATCGTCAAGTACAGTCC |
| LDHB-qR | ATCACGCGGTGTTTGGGTAAT |
| PDK1-qF | GAGAGCCACTATGGAACACCA |
| PDK1-qR | GGAGGTCTCAACACGAGGT |
| ACSL4-qF | AACCCAGAAAACTTGGGCATT |
| ACSL4-qR | GTCGGCCAGTAGAACCACT |
| SLC7A11-qF | ATGCAGTGGCAGTGACCTTT |
| **Primers** | **Sequences (5’- 3’)** |
| SLC7A11-qR | GGCAACAAAGATCGGAACTG |
| SLC3A2-qF | CTGGCTCCCTCCTTTCCTTG |
| SLC3A2-qR | TAGGAGAAGAGTCCAGGCCC |
