# Supplementary material for: Sulconazole Induces PANoptosis by Triggering Oxidative Stress and Inhibiting Glycolysis to Increase Radiosensitivity in Esophageal Cancer
Source: Mol Cell Proteomics. 2023 Apr 17;22(6):100551. doi: 10.1016/j.mcpro.2023.100551 (PMC10205543; doi:10.1016/j.mcpro.2023.100551)
Supplement: Supplementary Figure S1 [file mmc4.docx]

**Sulconazole induces PANoptosis by triggering oxidative stress and inhibiting glycolysis to increase radiosensitivity in esophageal cancer**

Lu-Xin Liu ^1,2^, Jing-Hua Heng ^1,2^, Dan-Xia Deng ^1,2^, Hui Zhao ^2,3^, Zhen-Yuan Zheng ^1,2,3^, Lian-Di Liao ^1,2^, Wan Lin ^1,3^, Xiu-E Xu ^1,2^, En-Min Li ^2*^, and Li-Yan Xu ^1,2,3*^

**Supplementary Figure Legend:**

**Fig. S1** **Sulconazole induces apoptosis, pyroptosis, necroptosis and ferroptosis.**

**
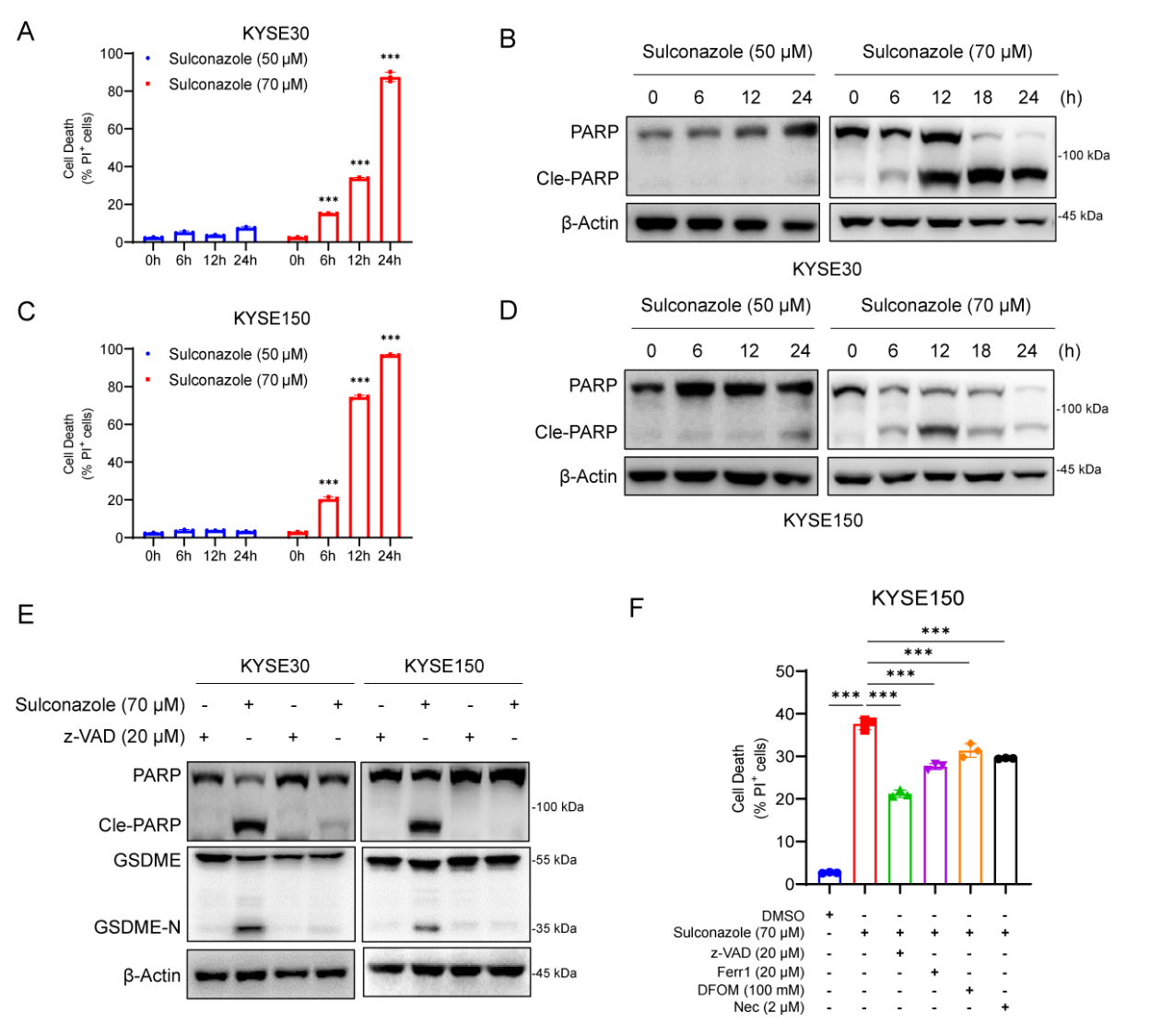
**

**Fig. S1** **Sulconazole induces apoptosis, pyroptosis, necroptosis and ferroptosis.** (**A, C**) Cell death of KYSE30 and KYSE150 cells subjected to the indicated treatments was detected by flow cytometry, and the numbers of PI-positive cells that may have undergone apoptosis, pyroptosis, necroptosis or ferroptosis were calculated and shown in diagrams. (**B, D**) Under the same conditions as (A, C), western blot detection of PARP and cleaved-PARP. (**E**) Western blot analyses of the expression of PARP, cleaved-PARP, GSDME and GSDME-N after sulconazole (70 μM) and z-VAD (20 μM) treatment for 12h in KYSE30 (left panel) and KYSE150 (right panel) cells. (**F**) Cell death measurement in KYSE150 cells treated with sulconazole (70 μM) for 6 hours and combined with various cell death inhibitors. Z-VAD (Z-VAD-FMK, 20 μM); Ferr1 (ferrostatin-1, 20 μM); DFOM (deferoxamine mesylate, 100 mM); Nec (necrostatin-1, 2μM). The data in (A, C and F) are representative of three independent experiments, and all are presented as the mean ± S.D. *P*-values were calculated by an unpaired two-sided Student’s *t*-test. **P* < 0.05, ***P* < 0.01, ****P* < 0.001.
