# Supplementary material for: Sulconazole Induces PANoptosis by Triggering Oxidative Stress and Inhibiting Glycolysis to Increase Radiosensitivity in Esophageal Cancer
Source: Mol Cell Proteomics. 2023 Apr 17;22(6):100551. doi: 10.1016/j.mcpro.2023.100551 (PMC10205543; doi:10.1016/j.mcpro.2023.100551)
Supplement: Original Western Blots [file mmc5.pdf]

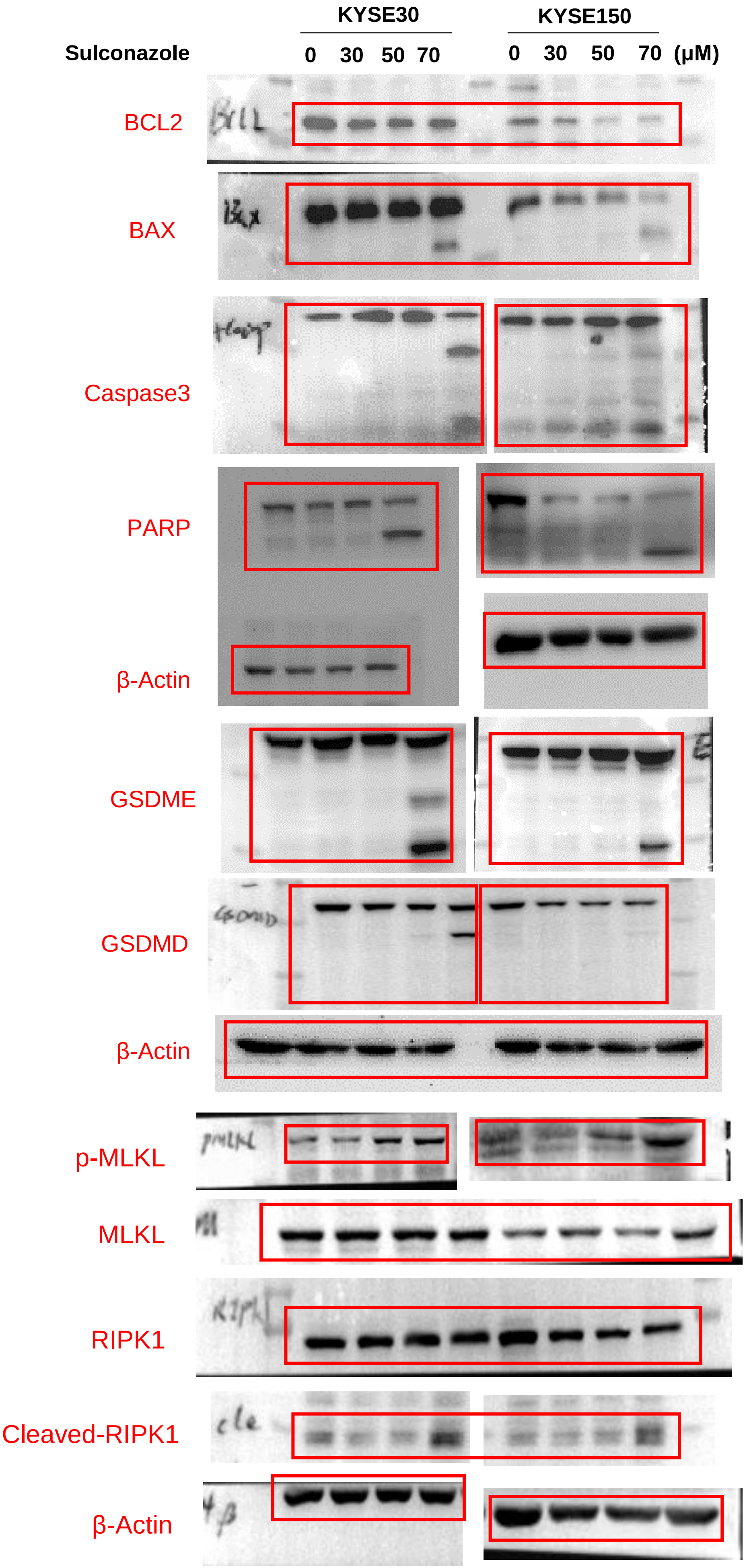

Figure S1. Uncropped Western Blot Images for Figure 4A.

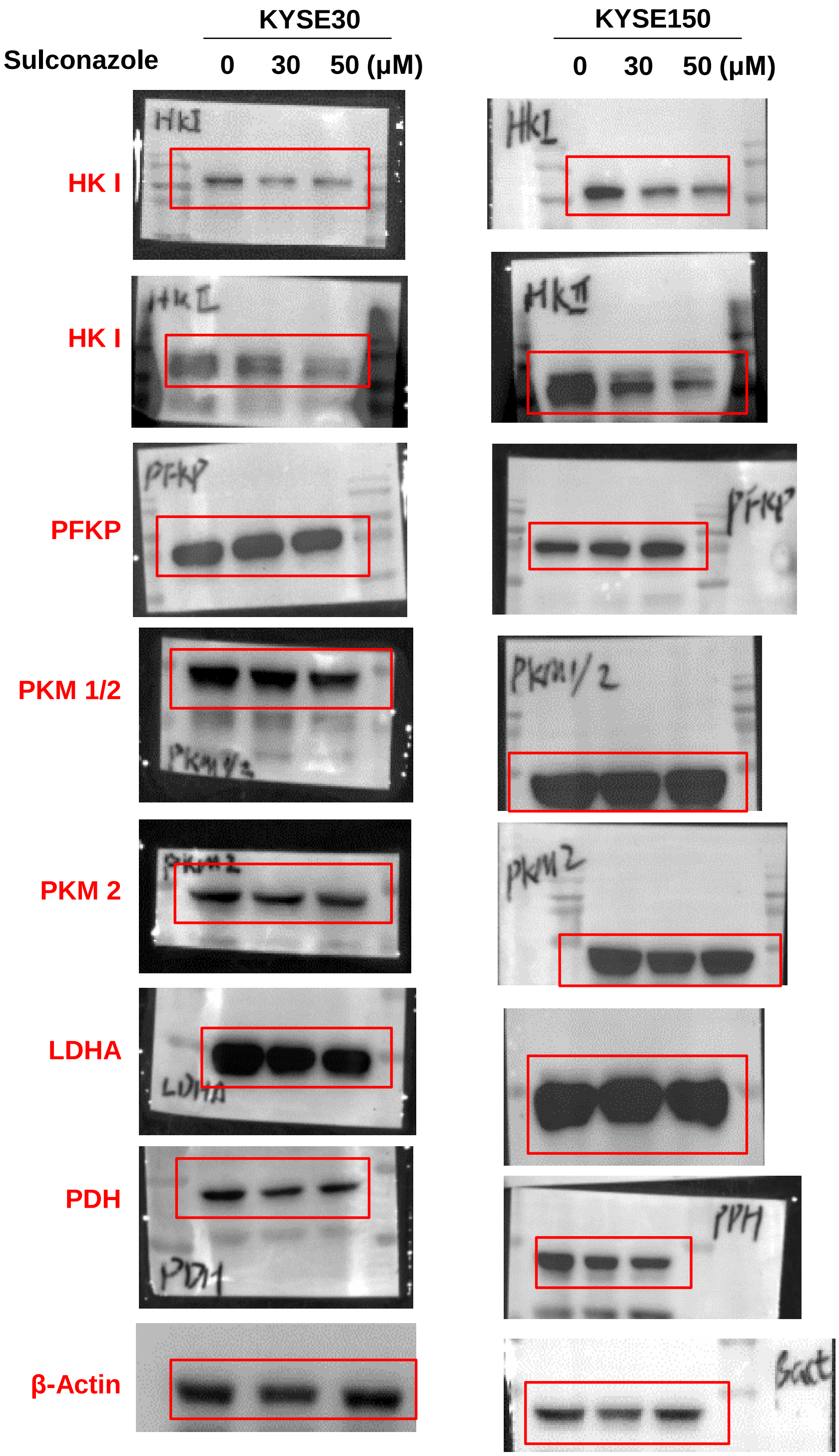

Figure S2. Uncropped Western Blot Images for Figure 5G.

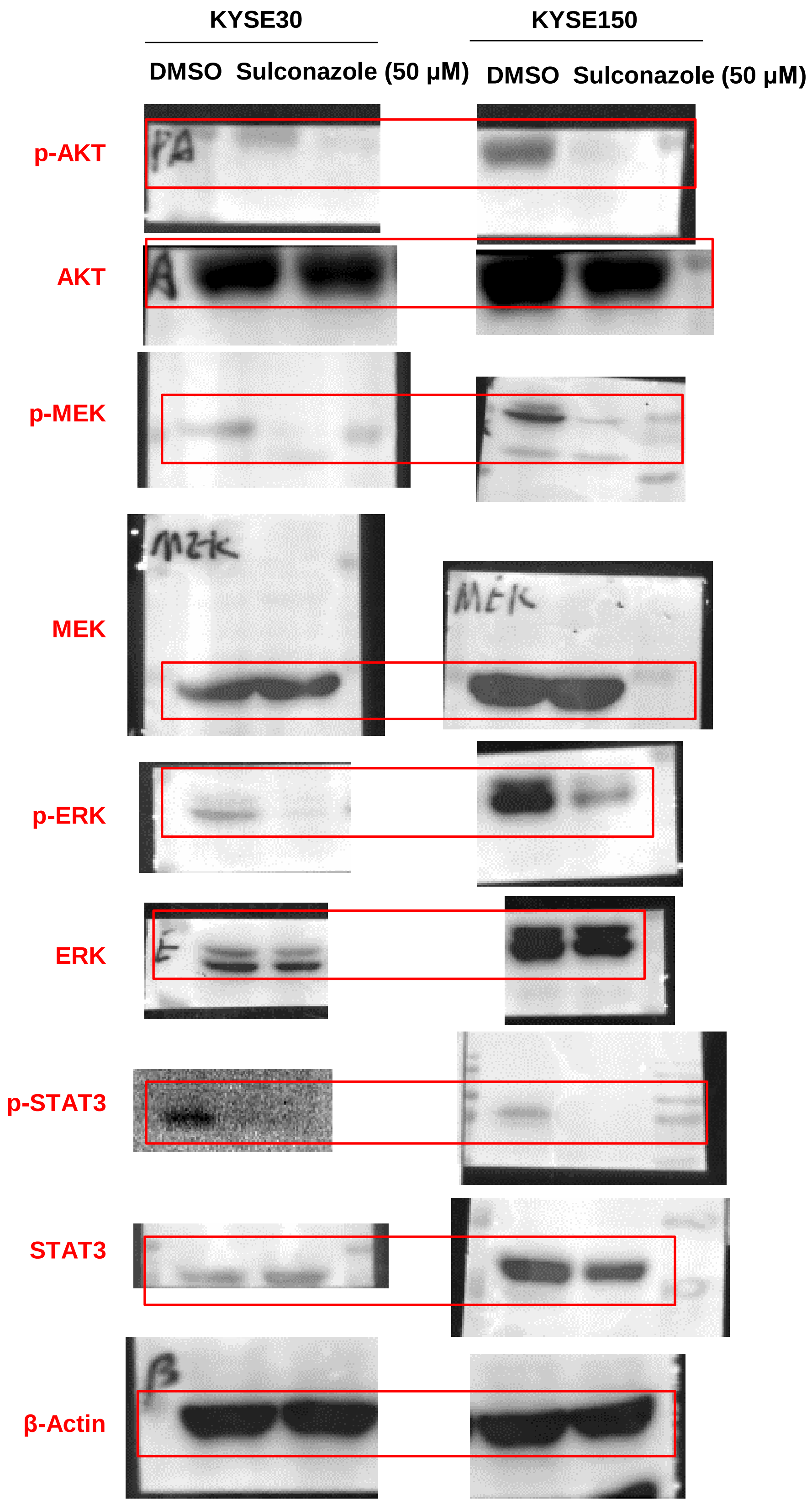

Figure S3. Uncropped Western Blot Images for Figure 5H.

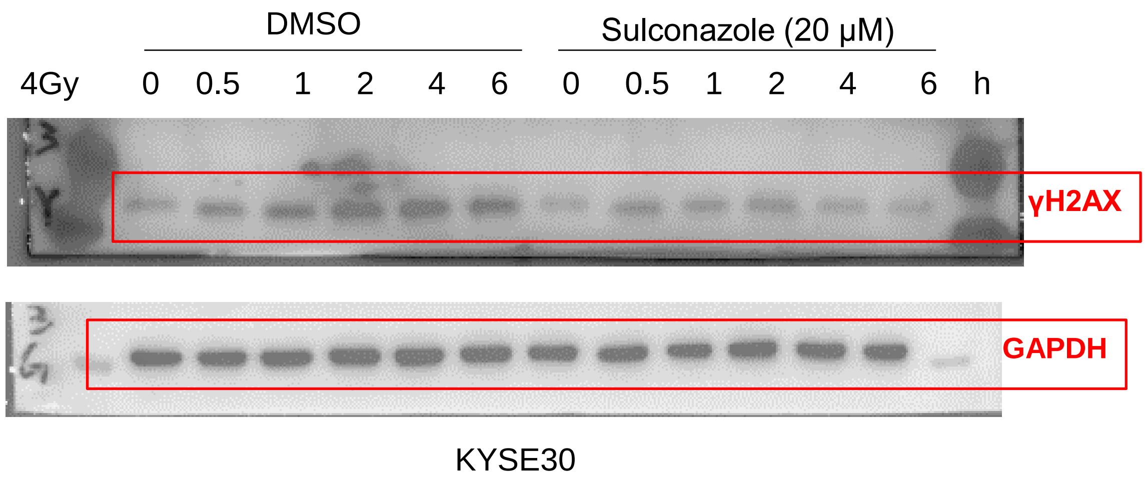

Figure S4. Uncropped Western Blot Images for Figure 6F.

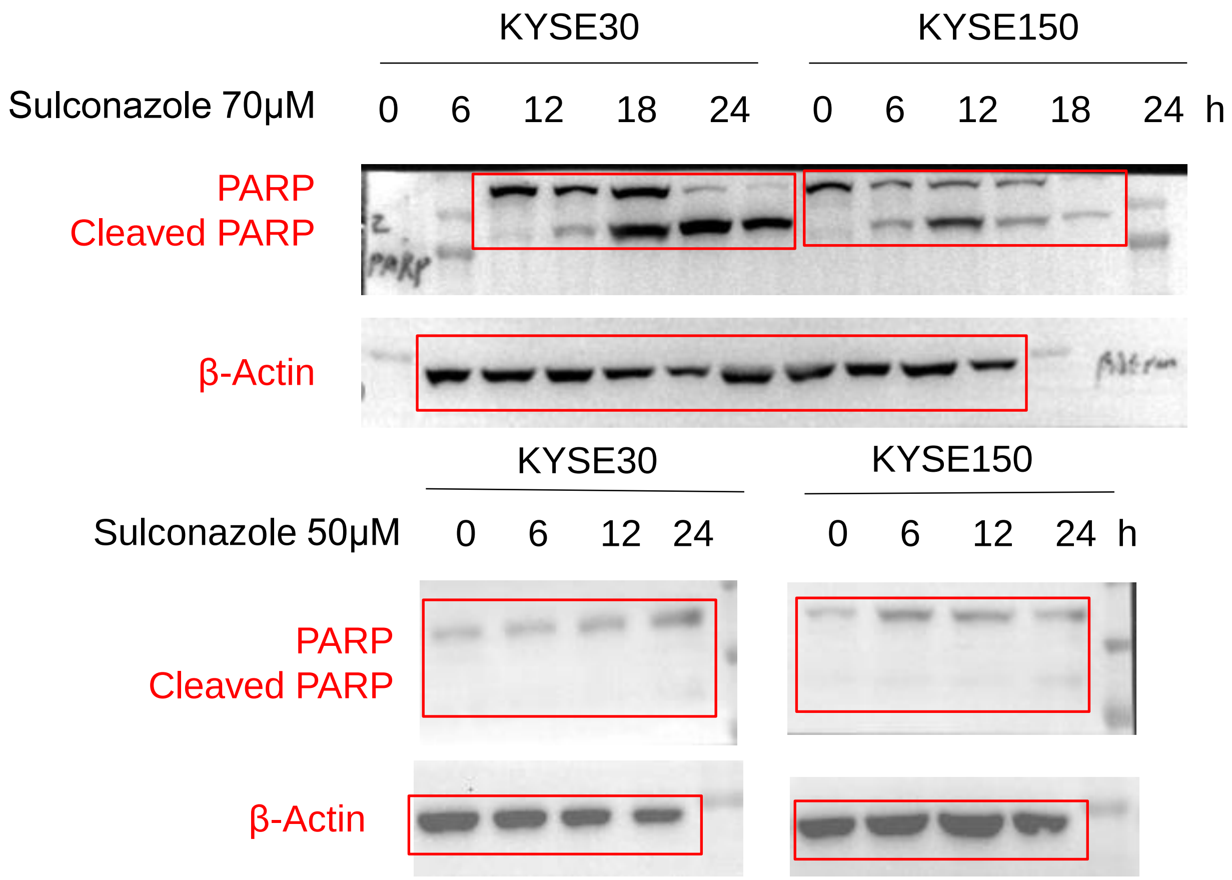

Figure S5. Uncropped Western Blot Images for Figure S1 B, D.

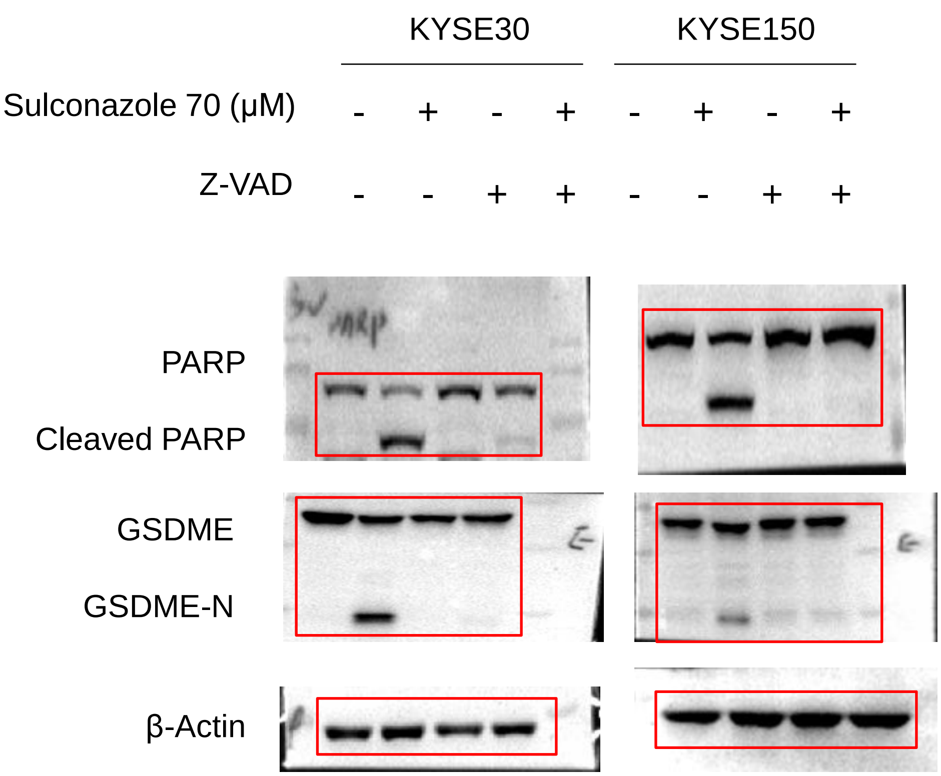

Figure S6. Uncropped Western Blot Images for Figure S1 E.
